# Vitamin D and parathyroid hormone in the umbilical cord blood – Correlation with light and dark maternal skin color

**DOI:** 10.1002/fsn3.3013

**Published:** 2022-08-05

**Authors:** Klara Wille, Aline Richard, Alexandra Nieters, Sabine Rohrmann, Katharina Quack Lötscher

**Affiliations:** ^1^ Department of Obstetrics University Hospital Zurich Zurich Switzerland; ^2^ Epidemiology Biostatistics and Prevention Institute, University of Zurich Zurich Switzerland; ^3^ FREEZE‐Biobank, Center for Chronic Immunodeficiency, Medical Center, Faculty of Medicine University of Freiburg Freiburg Germany

**Keywords:** parathyroid hormone, pregnancy, skin color, umbilical cord blood, vitamin D

## Abstract

During pregnancy, vitamin D deficiency is associated with negative health consequences for mother and child. Furthermore, dark skin color is associated with lower vitamin D levels. We investigated 25‐hydroxy‐vitamin D (25(OH)D) and parathyroid hormone (PTH) levels in mothers and in cord blood of their newborns depending on maternal skin color. We recruited 202 mother and child pairs at the University Hospital Zurich and measured 25(OH)D and PTH concentrations in maternal and postpartum umbilical cord blood. Skin type was self‐reported based on the Fitzpatrick Scale (type I to V). Uni‐ and multivariate methods were used to compare the maternal and neonatal 25(OH)D and PTH levels by skin type (light: I–III vs. dark: IV–V). As many as 54.5% of all mothers and 41.1% of the neonates were 25(OH)D deficient. This was higher in the neonates of dark‐skinned (55.9%) than in the neonates of light‐skinned mothers (38.1%; *p* = .06). The correlation of 25(OH)D in the maternal with umbilical cord blood was high (light: r = 0.85, dark: r = 0.87), with higher concentrations of 25(OH) vitamin D in the umbilical cord than in maternal blood. Regression analysis revealed that country of origin and maternal 25(OH)D concentration were the only statistically significant determinants for umbilical cord blood 25(OH)D. We observed no correlation of maternal with umbilical cord PTH concentrations; median PTH concentrations in the umbilical cord (5.6 pg/ml) were significantly lower than in maternal blood (25.7 pg/ml). The recommendation of vitamin D supplementation in newborns in their first 3 years of life should be particularly emphasized to dark‐skinned mothers.

## Graphical abstract

Our results showed that 54.5% of all mothers and 41.1% of all newborns were vitamin D deficient. In addition, we observed a strong positive correlation of vitamin D in all mother‐child pairs with levels of vitamin D in newborns, but there was no correlation in parathyroid hormone concentrations of mother and baby. The linear regression analysis revealed that South Asian, East Asian, or Pacific origin and the maternal vitamin D concentration were the only statistically significant determinants for umbilical cord blood vitamin D.

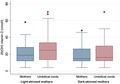

## 1 INTRODUCTION

An increasing number of studies on vitamin D are focusing on nonskeletal effects of vitamin D deficiency. There is evidence that vitamin D deficiency is associated with acute and chronic illnesses like infectious diseases (influenza, tuberculosis), type 2 diabetes mellitus, cardiovascular disease, depression, and cancer (Gruber‐Bzura, 2018; Lucas et al., 2008; Wacker & Holick, 2013). Low vitamin D levels are a worldwide public health problem, even in industrialized nations (Cashman et al., 2016; Hilger et al., 2014). An increase in the prevalence of rickets and osteomalacia is observed in countries such as Canada or England, particularly in dark‐skinned people (Goldacre et al., 2014; Uday & Högler, 2017). The prevalence of maternal vitamin D deficiency at or near term, defined as circulating 25‐hydroxy‐vitamin D [25(OH) vitamin D] <20 ng/ml, varies between 5% and 20% in light‐skinned populations and between 30% and 70% in dark‐skinned populations or persons wearing a veil (Cashman et al., 2016).

Associations between low maternal 25(OH) vitamin D concentrations and adverse perinatal outcomes such as gestational diabetes or hypertensive disorders have been described (Bärebring et al., 2016; Bodnar, Catov, et al., 2007; Yuan et al., 2021). As the fetus is dependent on the maternal pool of vitamin D, concern about the functional impact of hypovitaminosis D on the offspring in utero and later in life is growing (Dror & Allen, 2014). Concentrations of 25(OH) vitamin D in cord blood at delivery range from 68% to 108% of maternal concentrations (Kovacs, 2001, 2011). There are currently only few data assessing the vitamin D status of neonates in Switzerland (Krieger et al., 2018), and no 25(OH) vitamin D cut‐off values have yet been defined for newborns.

Vitamin D and parathyroid hormone (PTH) are calcium homeostatic hormones, regulating the absorption of calcium and phosphorus. Low vitamin D status ultimately leads to decreasing serum calcium levels, triggering the secretion of PTH, which stimulates bone resorption. During pregnancy, the higher calcium demand for the fetus, accreting about 30 g of calcium by term, is mainly covered by increased intestinal calcium absorption among several physiologic adaptations (Brannon & Picciano, 2011; Kovacs, 2001, 2016). Intestinal calcium absorption is doubled during pregnancy. This upregulation is mediated by 1,25‐dihydroxyvitamin D3 (calcitriol), prolactin, placental lactogen, and possibly by other factors (Kovacs, 2016; Evans et al., 2004). During a normal pregnancy, PTH concentration typically declines to low levels at the beginning of pregnancy, before returning to the midnormal range by term. This suppression may not occur, with subsequent secondary hyperparathyroidism, in pregnant women who have particularly low intakes of calcium and vitamin D (Kovacs, 2016). PTH cord blood concentrations are even lower than in maternal blood because it is suppressed by the hypercalcemia caused by active placental calcium transport (Bodnar, Catov, et al., 2007; Kovacs, 2001).

Variation of vitamin D levels by skin color in umbilical cord blood has not been examined in Switzerland. We investigated determinants of 25(OH) vitamin D in cord blood and the correlation of 25(OH) vitamin D and PTH levels in pregnant women and their neonates according to their skin color.

## 2 METHODS

### 2.1 Study population

Between September 2014 and June 2016, women with a planned cesarean section at 37 to 42 weeks of pregnancy were conveniently recruited at the University Hospital Zurich in the Clinic of Obstetrics. Informed consent was given. Maternal blood was collected within days prior to the cesarean section and venous umbilical cord blood was drawn within minutes after delivery together with a regular blood sample. The concentration in the umbilical cord blood was assumed to represent the variables' concentrations in the neonate.

The exclusion criteria of this study were pregnancy with multiples, below 18 years of age, known or suspected drug or alcohol abuse, HIV (human immunodeficiency virus), history of parathyroid, renal or liver disease, and chronic malabsorption syndromes or granuloma‐forming disorders. No participant reported use of diuretic medication or hormones influencing PTH metabolism. In total, we obtained data of 202 maternal and 185 cord blood samples.

### 2.2 Measurement of serum 25(OH) vitamin D and PTH

For the analysis of vitamin D and PTH, whole blood samples of 10 ml and 2.5 ml, respectively, were collected.

We measured the concentration of serum 25(OH) vitamin D, a marker commonly used to determine vitamin D status, within the same day of blood sampling at the Institute of Clinical Chemistry at the University Hospital Zurich, using the vitamin D total‐analysis Roche Cobas® electrochemiluminescence immunoassay (Roche Diagnostics). The detection range of this method is 3.0–70.0 ng/ml for 25(OH) vitamin D and has a variation coefficient of 2.2%–6.8%.

We defined vitamin D deficiency as 25(OH) vitamin D concentrations <20 ng/ml and sufficiency as ≥20 ng/ml following the recommendations of the Endocrine Society (Holick et al., 2011). For neonates or cord blood, no cut‐off values have been determined. Hence, we calculated the percentage of neonates with cord blood 25(OH) vitamin D concentrations <20 ng/ml, as stated in previous studies (Das et al., 2017; Eichholzer et al., 2013).

To determine PTH values, the 2.5 ml blood samples (ethylenediaminetetraacetic acid (EDTA) plasma) were analyzed using Elecsys® electrochemiluminescence immunoassay with the Roche Cobas e602 system. Measurement of intact PTH occurs by an extracting two‐site immunoradiometric assay (with two antibodies forming a sandwich complex being bound to an electrode) (Blind et al., 1987). The test has a variation coefficient of 1.3–3%. PTH concentrations between 15% and 65 ng/L are considered normal range for adult women (Flentje et al., 1990). A study defining pediatric reference values on another measuring system (IMMULITE®2000 by Samsung) suggested pediatric reference values of 10–112 ng/L PTH for neonates (Soldin et al., 2008).

### 2.3 Ethics statement

We performed the study according to the ethical principles in the Declaration of Helsinki and approved by the ethics committee of the canton of Zurich, Switzerland (KEK‐ZH‐Nr. 2013–0213). Written informed consent was given by each participant.

### 2.4 Skin color and other possible determinants of vitamin D status

In order to determine possible factors influencing vitamin D status, a questionnaire was filled in by each participant with the help of the treating physician as described previously (Richard et al., 2017). Besides skin color (dark vs. light), the following covariates were included: age, parity, body mass index (BMI) before pregnancy, weight gain during pregnancy, weeks of pregnancy, country of origin, season of blood collection (autumn, winter, spring, summer), frequency of sunscreen use during sun exposure in summer (never, sometimes, always), smoking status, and intake of supplements with vitamin D (yes vs. no). Regarding neonates, we assessed gestational age at birth, the 5‐minutes Apgar score (≥8 vs. < 8; ≥ 8 representing good adaptation) (American Academy of Pediatrics Committee on Fetus and Newborn and ACOG Committee of Obstetric Practice, 2017), weight at birth, body length, and head circumference.

Skin color was assessed using a five‐level scale (Richard et al., 2017) adapted from the Fitzpatrick classification (Fitzpatrick, 1988). The participants determined their skin type by choosing the one that best represents their skin color among five pictures and describing the reaction of the skin to sun exposure (when exposed in the early summer at noon for 45–60 min). When the physician's assessment disagreed with the participant's choice, the rounded arithmetic mean was used to determine the skin color type. Skin color variable was dichotomized into light skin color (Fitzpatrick levels I–III) and dark skin color (Fitzpatrick levels IV and V).

Categories for weight gain during pregnancy were based on the guidelines of the Institute of Medicine (IOM), depending on the prepregnancy BMI: one category for normal and low weight gain and one for high weight gain. The definition of normal weight gain is 11.5–16 kg at term for normal‐weight women (BMI 18.5–24.9 kg/m^2^), 7–11.5 kg for overweight women (BMI 25.0–29.9 kg/m^2^), and 5–9 kg for obese women (BMI ≥30.0 kg/m^2^) (Rasmussen & Yaktine, 2009). Underweight women were integrated with normal‐weight women due to their low number and are not further specified in this study.

The country of origin (place of mother's birth) was grouped into five categories: (I) Switzerland and Germany, (II) Northern America, Northern Europe, Caucasus, Central Asia, and New Zealand, (III) Southern Europe, Australia, Latin America, and the Caribbean, (IV) South Asian, East Asia, and Pacific, and (V) Africa and Middle East.

### 2.5 Statistical analyses

In the descriptive analyses, we included 202 women and 185 babies and cord blood samples, respectively. In the regression analysis, we included 185 complete mother–neonate pairs.

STATA software version 13.1 (College Station, TX, USA) was used for all statistical analyses. Geometric means and corresponding 95% confidence intervals (CIs) were calculated to examine differences in 25(OH)vitamin D and PTH concentrations between mothers with dark and light skin.

We performed descriptive statistics using Spearman correlation and Wilcoxon rank sum test to compare maternal and neonatal 25(OH) vitamin D and PTH levels depending on the dichotomized skin type (light: I–III vs. dark: IV–V). Using a linear regression model, we examined which factors were statistically significantly associated with 25(OH) vitamin D concentration in cord blood. We considered variables of clinical importance that have been shown to be determinants of 25 (OH) vitamin D concentration in previous studies (Krieger et al., 2018; Richard et al., 2017): maternal 25(OH) vitamin D and PTH concentrations, maternal age, country of origin, weight gain during pregnancy, intake of vitamin D‐containing supplements, season, sunscreen use during sun exposure in summer, and 5‐minutes Apgar score.

## 3 RESULTS

Table 1 provides baseline characteristics of mothers (*n* = 202) and neonates (*n* = 185). As many as 83.2% of all mothers had a lighter skin color (defined by Fitzpatrick scale I to III). We noted vitamin D deficiency in more than half of all mothers (54.5%) with a median serum 25(OH) vitamin D concentration of 18.0 ng/ml. Comparing vitamin D deficiency rate in light‐ and dark‐skinned mothers, no statistically significant difference (light: 52.4% vs. dark: 64.7%) was detected (*p* = .6). In 41.1% of umbilical cord samples, we observed deficient vitamin D levels. Median 25(OH) vitamin D concentration in the umbilical cord of dark‐skinned mothers was lower with 16.0 ng/ml compared to 24.7 ng/ml in umbilical cord of light‐skinned mothers, but this difference was not statistically significant (*p* = .12). Deficiency rates in neonates of light‐skinned mothers were lower than those of dark‐skinned mothers (light: 38.1% vs. dark: 55.9%; *p* = .06).

**TABLE 1:** General characteristics of mothers and their neonates with light and dark skin color

Variables of interest	Light skin color^a^	Dark skin color^b^	Total
Mothers			
*n* (%)	168 (83.2)	34 (16.8)	202 (100)
25(OH)D ng/ml, median (Q1, Q3)	18.8 (11.9; 28.1)	14.6 (11.9; 26.1)	18.0 (11.9; 28.1)
Parathyroid hormone ng/L, median (Q1, Q3)	25.4 (18.5; 31.6)	27.5 (21.2; 38.2)	25.7 (18.8; 36.3)
Age, mean (SD)	33.7 (5.2)	32.9 (4.3)	33.6 (5.1)
Parity (% Multiparae)	67.9	82.3	70.3
BMI before pregnancy kg/m^2^, median (Q1, Q3)	22.9 (21.2; 25.4)	25.2 (22.1; 27.9)	23.2 (21.2; 25.7)
Weight gain in pregnancy according to IOM^c^, %			
Normal and low	56.6	52.9	55.9
High	33.3	41.2	34.7
Missings	10.1	5.9	9.4
Country of origin, %			
Switzerland and Germany	38.9	5.9	33.3
Northern America, Northern Europe, Caucasus, Central Asia, and New Zealand	35.3	8.8	30.8
South Europe, Australia, Latin America, and the Caribbean	15.0	14.7	14.9
South‐, East Asia and Pacific	5.4	26.5	9.0
Africa and Middle East	5.4	44.1	11.9
Smoking status, %			
Never smoker	61.1	55.9	58.5
Ever smoker	29.3	35.3	32.3
Current smoker	9.6	8.8	9.2
Intake of vitamin D‐containing supplements, %			
Yes	77.6	75.0	77.1
No	22.4	25.0	22.9
Season^d^, %			
Winter	31.0	26.5	28.7
Spring	39.3	35.3	37.3
Summer	8.9	11.8	10.3
Fall	20.8	26.5	23.6
Using sun protection in summer, %			
Never	15.5	47.1	31.3
Sometimes	42.3	29.4	35.8
Always	42.3	23.5	32.9
Neonates			
*n* (%)	154 (83.2)	31 (16.8)	185
25(OH)D ng/ml, median (Q1, Q3)	24.7 (13.2; 33.5)	16.0 (12.1; 29.7)	23.6 (12.8; 33.2)
Parathyroid hormone ng/L, median (Q1, Q3)	5.6 (4.7; 6.9)	5.9 (4.4; 8.3)	5.6 (4.7; 7)
Gestational age at birth, mean (CI 95%)	38 (38; 38)	38 (38; 38)	38 (38; 38)
Good postnatal adaptation: 5 minutes Apgar score ≥8 (%)	97.6	94.1	97.0
Weight at birth (g), mean (CI 95%)	3317 (3254–3380)	3205 (3085–3324)	3298 (3242–3354)
Length at birth (cm), mean (CI 95%)	49.3 (49.0–49.6)	48.7 (48.0–49.3)	49.2 (48.9–49.4)
Head circumference (cm), mean (CI 95%)	35.1 (34.9–35.3)	34.6 (34.3–34.9)	35.0 (34.8–35.2)

^a^ Light skin color was defined as category I to III from the Fitzpatrick scale.

^b^ Dark skin color was defined as category IV to V from the Fitzpatrick scale.

^c^ Weight gain during pregnancy was split into normal, low, and high weight gain according to the Institute of Medicine (IOM) guidelines.

^d^ Seasons were divided into winter (December 21–March 20), spring (March 21–June 20), summer (June 21–September 20), and autumn (September 21–December 20).

**FIGURE 1:**
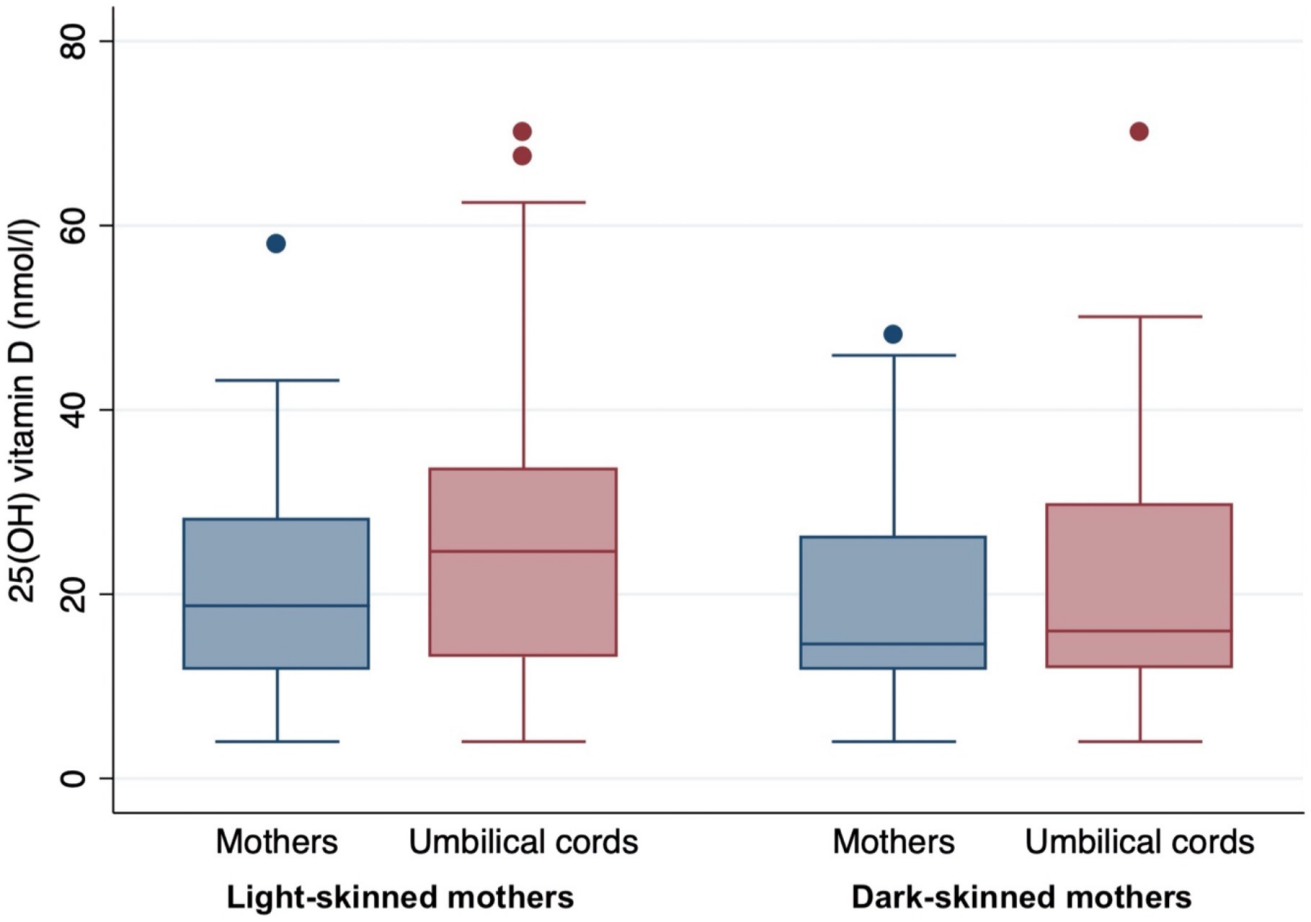
Concentration of 25(OH) vitamin D in maternal and umbilical cord blood for light‐skinned and dark‐skinned mothers. The boxplots lower limit indicates Q1, the upper limit Q3, and the horizontal line the median (Q2). The whiskers show minimal and maximal values, while dots indicate statical outliers (corresponding to 1.5 times of the interquartile range).

Correlation of cord blood 25(OH) vitamin D with maternal blood was high in both skin‐color groups (light: r = 0.85, dark: r = 0.87), with higher concentrations of 25(OH) vitamin D in umbilical cord than in maternal blood (Figure 1).

Parathyroid hormone (PTH) levels above 65 ng/L, corresponding to hyperparathyroidism, were observed in 1.5% of all mothers (1.2% in light‐skinned vs. 2.9% in dark‐skinned; *p* = .44). Low PTH levels (< 15 ng/L) affected 10.9% of all mothers (11.3% in light‐skinned vs. 8.8% in dark‐skinned; *p* = .67). Median serum concentration of PTH was 25.4 ng/L in light‐skinned and 27.5 ng/L in dark‐skinned mothers, *p* = .35.

**FIGURE 2:**
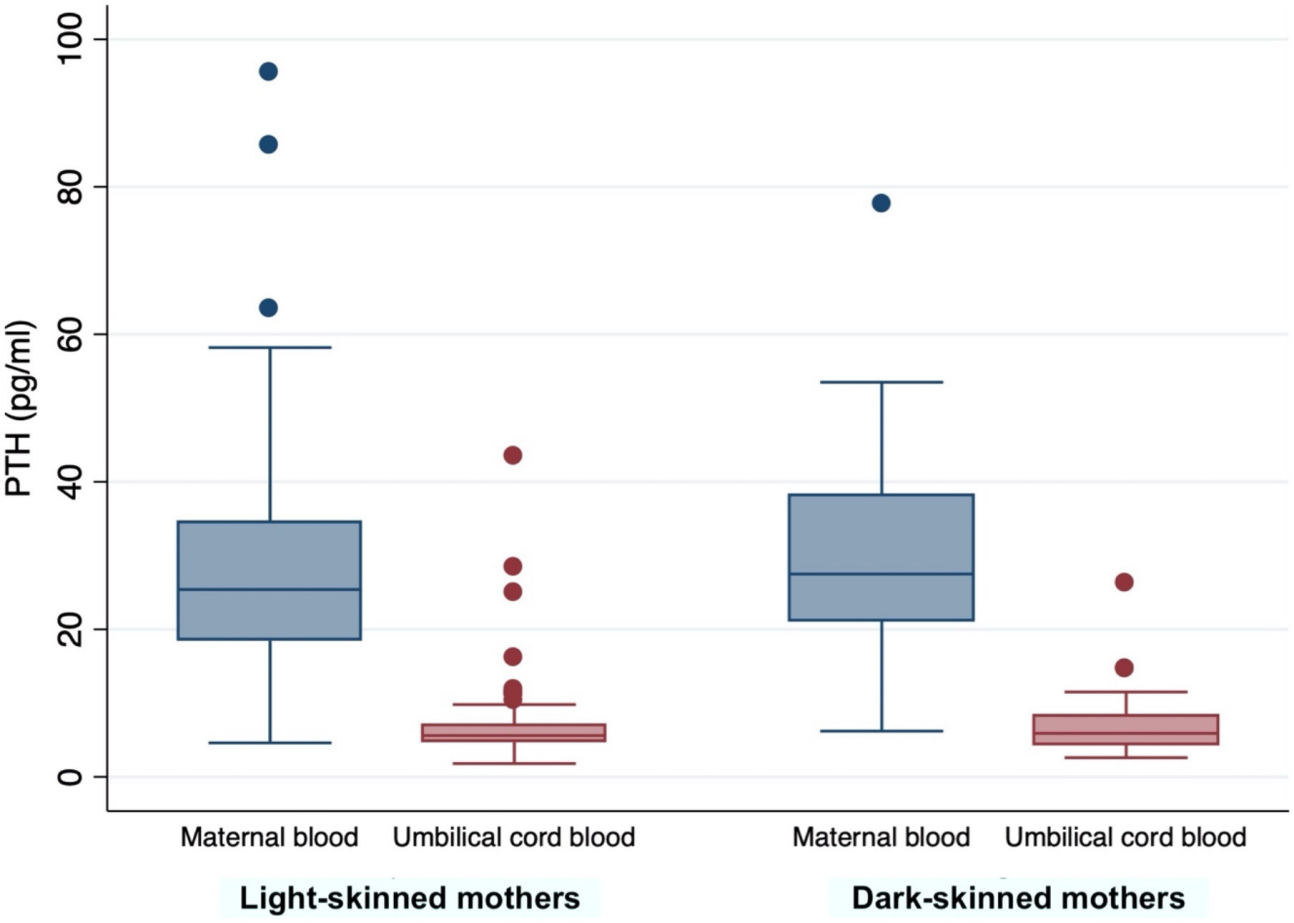
Concentration of parathyroid hormone (PTH) concentrations in maternal and umbilical cord blood for light‐skinned and dark‐skinned mothers. The boxplots lower limit indicates Q1, the upper limit Q3, and the horizontal line the median (Q2). The whiskers show minimal and maximal values, while dots indicate statical outliers (corresponding to 1.5 times of the interquartile range).

We observed significantly lower median PTH concentrations in the umbilical cord than in maternal blood, regardless of skin color (both *p*‐values <.005; Figure 2). The group with deficient 25(OH) vitamin D cord blood levels had comparable median PTH concentrations in the cord blood (5.9 ng/L, Q1 4.7; Q3 7.0) to the nondeficient group (5.4 ng/L, Q1 4.5; Q3 7.0). PTH levels in neonates did not significantly correlate with maternal levels (r = −0.02, *p* = .78). The negative correlation between 25(OH) vitamin D and PTH in pregnant women (r = −0.30, *p* < .0001) was not observed in the umbilical cord blood (r = −0.06, *p* = .44).

Being of South Asian, East Asian, or Pacific origin and maternal 25(OH) vitamin D concentration were the only statistically significant determinants of umbilical cord blood 25(OH) vitamin D. A higher level of maternal 25(OH) vitamin D was significantly associated with a higher level of 25(OH)vitamin D in the umbilical cord, and neonates of mothers from South Asian, East Asian, or Pacific origin had lower 25(OH) vitamin D concentrations compared to neonates of Swiss mothers (Table 2).

**TABLE 2:** Multivariable regression analysis and determinants for vitamin D levels in cord blood

Variables of interest	Coef.	[95% CI]	
Maternal vitamin D	**0.868**	**0.780**	**0.955**
Maternal PTH	−0.067	−0.167	0.032
Skin color			
Light	Ref.		
Dark	−0.070	−0.235	0.095
Age	0.006	−0.005	0.016
Country of origin			
Switzerland and Germany	Ref.		
Northern America, Northern Europe, Caucasus, Central Asia, and New Zealand (without Switzerland and Germany)	−0.023	−0.142	0.095
Southern Europe, Australia, Latin America, and the Caribbean	−0.077	−0.231	0.077
South‐ and East Asia and Pacific	**−0.202**	**−0.395**	**−0.008**
Africa and Middle East	−0.157	−0.362	0.047
Weight gain in pregnancy according to IOM			
Normal and Low	Ref.		
High	0.020	−0.080	0.120
Missings	0.161	−0.009	0.330
Smoking status			
Never smoker	Ref.		
Ever smoker	−0.007	−0.114	0.099
Current smoker	−0.101	−0.276	0.073
Intake of vitamin D‐containing supplements			
No	Ref.		
Yes	0.085	−0.027	0.197
Season			
Autumn	Ref.		
Winter	−0.068	−0.199	0.063
Spring	−0.057	−0.180	0.067
Summer	−0.161	−0.340	0.019
Sunscreen use during sun exposure in summer			
Never	Ref.		
Sometimes	0.016	−0.123	0.154
Always	0.002	−0.143	0.147
5 min Apgar score			
≥8	Ref.		
<8	0.057	−0.263	0.377

## 4 DISCUSSION

In this study, 41.1% of all neonates had deficient 25(OH) vitamin D concentrations. Neonates of dark‐skinned mothers were more frequently affected than neonates of light‐skinned mothers (55.9% vs. 38.1%). 25(OH) vitamin D concentrations were consistently higher in the umbilical cord blood than in maternal blood, and we observed a high correlation of 25(OH) vitamin D between cord blood and maternal blood in both skin‐color groups. The latter corresponds with findings in previous studies, which show a strong correlation between the concentrations of 25(OH) vitamin D in cord blood and the maternal concentration (Bassir et al., 2001; Bodnar, Simhan, et al., 2007; Krieger et al., 2018; Rabbani et al., 2021; Wegienka et al., 2016). But in all except two of these studies, cord blood 25(OH) vitamin D levels were lower compared to the maternal levels of vitamin D, which contrasts with our results; Wang et al. (2010) and Nicolaidou et al. (2006) detected higher 25(OH) vitamin D levels in umbilical cord blood.

In accordance with our results, Wegienka et al. (2016) observed that white neonates and their mothers had a trend for higher 25(OH) vitamin D levels compared to black children and their mothers. In their study, 73% of the 241 maternal–child pairs were black. Maternal levels were measured at study enrollment during the third trimester of pregnancy and the level of the child was measured at birth in the umbilical cord. Similar results were reported in two other studies (Bodnar, Simhan, et al., 2007; Hollis & Pittard, 1984). On the other hand, Rabbani et al. (2021) did not observe any difference in 25(OH) vitamin D levels in the 213 maternal–child pairs with respect to skin color.

Eichholzer et al. (2013) observed among 113 male neonates that unadjusted mean 25(OH) vitamin D concentrations were significantly lower in black neonates (11.44 ng/mL) than in white neonates (18.24 ng/ml). The percentage of deficient 25(OH) vitamin D levels was much higher in black (84%) than in white (63%) neonates (Eichholzer et al., 2013). Uday et al. (2017) analyzed vitamin D deficiency and insufficiency (defined as 25(OH) vitamin D <12 ng/ml and <20 ng/ml, respectively) on dried blood spots of 2999 newborn. Nearly 70% of neonates had 25(OH) vitamin D levels <20 ng/ml, with the lowest median levels in black and Asian ethnic groups. In neonates of Asian, black, and mixed ethnicities, the proportion of deficiency was also higher (Uday et al., 2017).

According to the regression model, the main determinant of 25(OH) vitamin D level in cord blood is the maternal 25(OH) vitamin D level. Another determinant is the maternal origin from South Asia, East Asia, and the Pacific region. This finding is similar to those of other studies, which reported lower serum vitamin D in cord blood of UK‐born Asians compared to that of Whites (Okonofua et al., 1987; Sulaiman et al., 2010). Cultural difference in clothing, mainly wearing a veil, might contribute to these differences. Contrary to other studies (Bodnar, Simhan, et al., 2007; Bowyer et al., 2009; Eichholzer et al., 2013), other possible determinants of vitamin D levels such as season of birth, vitamin D supplementation, prepregnancy BMI, and maternal age were not statistically significant in our study. Similar to our results, Rhabbani et al. failed to find any statistically significant association between characteristics of the mother (age, BMI, parity, and sunlight exposure) or their newborns (weight, length, and head circumference) with 25(OH) vitamin D deficiency (Rabbani et al., 2021).

Maternal PTH levels were in most cases within the normal range. Only 1.5% had levels above the upper limit (PTH > 65 ng/L) and 10.9% low levels (PTH < 15 ng/L). This is not what one would expect in the situation of low 25 (OH) vitamin D levels. It has been described that PTH is lower in pregnant than in nonpregnant women (Saxe et al., 1997); therefore, the upper reference value of normal PTH may be too high in the pregnant population. This could explain the absence of secondary hyperparathyroidism in vitamin‐D deficient pregnant women. Bowyer et al. (2009) reported elevated concentrations of PTH (≥5 ρmol/L equivalent to 47 ng/L) in 75% of vitamin‐D deficient (25 (OH) vitamin D < 10 ng/ml) and in 14% of vitamin‐D insufficient (10 to <20 ng/ml) pregnant women.

In accordance with other studies, we observed considerably lower PTH levels in umbilical cord blood than in maternal blood. Cord blood PTH concentrations in the vitamin‐D deficient group did not differ significantly from PTH levels in the nondeficient group. Bowyer et al. reported a median maternal PTH level of 1.7 ρmol/L (16 ng/L) in contrast to the median cord blood PTH level of 0.2 ρmol/l (1.9 ng/L) (Bowyer et al., 2009). Contrary to our results, PTH concentration was elevated in 5% of vitamin‐D deficient and 1% of insufficient neonates (Bowyer et al., 2009). Saxe et al. also found suppressed PTH levels in cord blood (Saxe et al., 1997). Sulaiman et al. compared white and South Asian neonates born in the UK, showing low PTH levels in both groups with statistically significant higher PTH concentrations in South Asian neonates in comparison to white neonates (Sulaiman et al., 2010).

In our study, PTH concentration in umbilical cord blood was not associated with maternal PTH concentration, probably due to alternative mechanisms for calcium exchange. Additionally, the immaturity of the fetal parathyroid glands is an established phenomenon. Kovacs et al. showed that elevated calcium levels in the fetus, caused by active placental calcium transport, result in a suppression of fetal PTH production (Kovacs, 2001). New insights into the mechanisms of maternal–fetal mineral homeostasis have recently been established. Fetal calcium concentrations are regulated by the calcium sensing receptor (CaSR) being primarily expressed in the fetal parathyroid gland and kidney. By the 10th week of gestation, PTH is synthesized by the fetal parathyroid gland, but circulating concentrations are low during fetal life due to relative hypercalcemia dictated by the CaSR receptor (Taylor‐Miller & Allgrove, 2021).

A strength of our study is the variety of countries of origin of the participating women resulting in different skin pigmentation. Additionally, a variety of confounders were considered. However, some possible determinants of vitamin D and PTH levels were not assessed, such as physical activity and serum calcium concentration. Women participating in our study came from the general population in Zurich; however, as participants were recruited only from one hospital, our results are not generalizable to other populations. In our study, dosing of vitamin D supplements was not available. But according to Table 1, there was no difference in supplement intake in both groups. The general recommendation in Switzerland for pregnant women is to supplement 600–800 IE of vitamin D during pregnancy. However, we do not know whether women take the recommended dose regularly. Lastly, we were not able to recruit an equal number of women with light and dark skin color and, therefore, were not able to detect smaller differences between the two groups.

## 5 CONCLUSIONS

There is growing interest in investigating ethnic variation of vitamin D during pregnancy because of the consequences on maternal as well as child health. To the best of our knowledge, cord blood vitamin D and PTH have not yet been assessed by maternal skin color in Switzerland. Although vitamin D cord blood concentrations tend to be higher than maternal levels, the prevalence of vitamin D deficiency in umbilical cord is high, with an even higher risk for newborns of dark‐skinned mothers as well as mothers coming from South Asia, East Asia, and the Pacific area. Based on these findings, further studies are needed to confirm these associations, and, in the future, health care professionals should be advised to recommend vitamin D supplementation in newborns in their first 3 years of life, particularly for newborns of dark‐skinned mothers and mothers from South Asia, East Asia, and the Pacific area.

## ACKNOWLEDGEMENT

We thank all women who participated in this study and Nina Pupikofer for data entry.

## FUNDING INFORMATION

This study was funded by the Swiss National Science Foundation (NRP69 grant 4069–145194). The Swiss National Science Foundation had no role in the design, analysis, or writing of this article.

## CONFLICT OF INTEREST

The authors declare that they have no competing interests.

## Data Availability

The data sets used and analyzed during the current study will be made available upon reasonable request made through the corresponding author.
